# A Survey on the Security of Low Power Wide Area Networks: Threats, Challenges, and Potential Solutions

**DOI:** 10.3390/s20205800

**Published:** 2020-10-14

**Authors:** Kuburat Oyeranti Adefemi Alimi, Khmaies Ouahada, Adnan M. Abu-Mahfouz, Suvendi Rimer

**Affiliations:** 1 Department of Electrical & Electronic Engineering Science, University of Johannesburg, Johannesburg 2006, South Africa; kouahada@uj.ac.za (K.O.); a.abumahfouz@ieee.org (A.M.A.-M.); suvendir@nedbank.co.za (S.R.); 2 Council for Scientific and Industrial Research (CSIR), Pretoria 0184, South Africa

**Keywords:** attacks, CIA triad, Internet of Things (IoT), low power wide area network, security, sigfox

## Abstract

Low power wide area network (LPWAN) is among the fastest growing networks in Internet of Things (IoT) technologies. Owing to varieties of outstanding features which include long range communication and low power consumption, LPWANs are fast becoming the most widely deployed connectivity standards in IoT domain. However, this promising network are exposed to various security and privacy threats and challenges. For reliable connectivity within networks, the security and privacy challenges need to be effectively addressed with proper mitigation protocol in place. In this paper, a comprehensive review on the security feature of LPWAN is presented. The paper mainly focuses on analyzing LPWAN’s key cybersecurity architecture and it present a significant emphasis on how the LPWAN is highly attractive to intruders and attackers. This paper aims at summarizing recent research works on key LPWAN security challenges such as replay attack, denial-of-service attack, worm hole attack, and eavesdropping attack, the effect of the attacks, and most importantly the various approaches proposed in the literature for the attacks’ mitigation. The paper concludes by highlighting major research gaps and future directions for the successful deployment of LPWAN.

## 1. Introduction

The evolutionary trends in communication technologies which brought about inventions which includes the Internet of Things (IoT) and Internet of Everything (IoE) have substantially created new and better living standards for humans and their environments [1,2]. By enabling efficient communication between varieties of devices, the IoT technologies are playing crucial roles in enhancing human quality of living in a broad spectrum of domains that are not limited to day to day smart applications (smart homes, smart transportation, smart education, smart cities), economy applications (enhancement of productivity in manufacturing plants, mining fields, oil and gas fields), health care applications, and security applications [3,4,5,6,7]. Arguably the fastest growing field in the history of computing, IoT is constantly bringing radical changes to human lives [8,9]. It is estimated that about 28 billion machine-to-machine (M2M) and consumer electronic devices are projected to be communicating over short-range radio technologies by 2021. Also, by 2024, IoT applications are expected to accrue a revenue of 4.3 trillion dollars [10]. Varieties of communication approaches and connectivity standards have been deployed in IoT environment. Popular short-range standards such as IEEE 802.15.1 and IEEE 802.15.4 have been widely used but their short communication range have been well publicized as a major limitation especially as various key IoT domains require extensive communication range. As alternatives, cellular networks which allows wide connectivity range have been extensively deployed in various capacities. However, the issue of cost and complexity is a major constraint. Low power wide area networks (LPWAN) have presented a viable option to the various shortcomings of the existing IoT connectivity standards.

As one of the most prominent IoT connectivity inventions in recent years, LPWAN is a wireless telecommunication technology with enviable characteristic features which includes long-range coverage, low data rates, low power consumption, and low-cost end devices. Also, they have the capacity to accommodate numerous end devices, long battery life, and adaptation to licensed and unlicensed spectrum, and most of the standards utilize simplified star network topology [10,11,12,13]. The authors in [13] categorized capacity and densification, energy efficient operations, coverage, location identification, security and privacy, cost-effectiveness, traffic characteristics etc. as key characteristics and requirements for LPWAN standards. As such, several LPWAN technologies, which include Long Range (LoRa), Random Phase Multiple Access (RPMA), Narrowband Internet of Things (NB-IoT), Sigfox, and Weightless, have been developed and deployed in various capacities. Each LPWAN standard has distinctive operational features which makes them suitable for varieties of tasks and operational environments.

However, despite the desirable features that the LPWAN technologies possess, security and privacy concerns have been a major challenge to its wide-range deployment. Due to the heterogeneity, ubiquity, and easy accessibility to devices in the network, LPWAN vulnerabilities to threats and intrusion have continued to rise geometrically [5]. Also, the fact that LPWAN standards mostly deploy simple protocols for bootstrapping and authentication, security and privacy due to their low cost and low energy consumption tags can be ascribed as a major reason for their security vulnerabilities and exploitable threats. Numerous cyberattacks and intrusion menaces in the form of eavesdropping, replay attack, denial of service (DoS) attacks, etc. have been major issues targeting the successful deployment of LPWAN. Major topics such as key management and storage, bootstrapping and authentication have been an area that have continued to be addressed in current research work. Keys can be compromised, devices with weak malware protections can be used as “bots” to forward malicious codes to infest other devices and the entire network can be intruded on. A recent HP study reveals that 70% of IoT devices are vulnerable to various forms of cyberattacks [14]. Thus, strong encryption and authentication of the application payload and the network admission request, etc. are some of the methodology proposed in the literature. The threats, effects and impacts of LPWAN security menaces have been extensively discussed in numerous studies [15,16,17,18]. To mitigate the growing IoT and LPWAN security challenges, numerous security solutions such as intrusion detection systems (IDSs), key management and encryption schemes have been proposed in the literature [19,20,21,22]. Despite the satisfactory performances demonstrated, some of the various schemes and upgrades from the standard producers have struggled in addressing the emerging security requirements of the IoT operational environment, e.g., despite LoRaWAN’s specification that laid emphasis on key management protocol whereby a compromised key from an end node should not create a major problem for other nodes, experiences have shown that key management issues is still a major problem with regards to the standard. Thus, there is a need to present efficient and effective security solutions that can urgently detect and mitigate LPWAN attacks and vulnerabilities. Focusing on analyzing LPWAN’s key cybersecurity architectural framework and technical features, a comprehensive review of the most recent research works that discussed the security aspect of LPWAN is presented in this paper. The paper aims at summarizing recent research methodologies on key LPWAN security challenges and varieties of threats, the effect of the attacks, and most importantly the various approaches proposed in the literature for the attacks’ mitigations. The main contributions of the paper are stated briefly as follows:

- We present a comprehensive review of the security and privacy issues and attacks that affect LPWAN. The attacks are categorized under the prominent information security requirements known as Confidentiality, Integrity, and Availability (CIA triad);
- a detailed analysis of the different techniques and security solutions that have been proposed in the literature for securing LPWAN are extensively discussed. The analysis entails a comparison of the mitigation methodologies, the types of attacks mitigated, the security requirements and limitations; and
- challenges and research gap in existing LPWAN security methods and the direction for future works are broadly analyzed.

Accordingly, the reminder of the paper is organized as follows: The related works on IoT and LPWAN is presented in Section 2. Section 3 presents an overview of the LPWAN. Section 4 presents the LPWAN technologies. Section 5 presents a brief security analysis of LPWAN. The section discusses the vulnerabilities and threats attributed to LPWAN. In Section 6, we discuss and compare some of the proposed techniques in the literature for mitigating attacks in LPWAN. The research challenges and recommendations for future works are discussed in Section 7. Lastly, Section 8 presents the conclusion. 

## 2. Related Survey Papers

In line with the increasing concerns regarding LPWAN and IoT security vulnerabilities, several survey works have presented detailed description of LPWAN challenges and mitigation methodologies. This section presents some of the recent works in the literature.

The authors in [15,18] presented a brief survey on LPWAN, specifically on LoRaWAN security. The authors discussed some possible attacks in the LPWAN and mentioned a few solutions for their mitigation. Miller [23] presented a survey on security issues precisely on LoRa and LoRaWAN. The author emphasized that the prevention of LoRa and LoRaWAN attacks require the application of good key management practices. By focusing on key management problems, the author suggested that efficient solutions need to be developed in order to prevent the end user from cyberattacks and hacking. The authors in [24] presented a survey on the deployment of LoRaWAN for IoT communication focusing on the physical and network layer of the LPWAN standard. The authors identified some LoRaWAN challenges, possible improvements and extensions to the LoRa and LoRaWAN standard. In a similar LoRaWAN study, the authors in [16] presented a security risk analysis of LoRaWAN using European Telecommunications Standards Institute (ETSI) guidelines. The work analyzes LoRaWAN v1.1 threat scales and impacts. 

The authors in [25] conducted a detailed survey of IoT security. The authors suggested that software defined network (SDN) and block chain should be employed to address the issue of key management, confidentiality, integrity, and privacy in IoT systems. They highlighted the benefits that blockchain and SDN can bring to the IoT security in terms of flexibility and scalability. The article only focused on SDN and blockchain domain. Similarly, the authors in [26] presented major security issues in IoT. The authors discussed and categorized security attacks based on the IoT layered architecture. They briefly discussed the mechanisms suggested in the literature for leveraging IoT security at different levels. Furthermore, the authors referred to some open research challenges in IoT security. The work presented in [27] focused on various attacks that can take place on different layers of IoT. The authors discussed some security mechanisms which can detect and prevent the attacks. In a similar study, the authors in [28] presented a survey on security and privacy issues in IoT architectural layers (the perception, network, transport, and application layer). The authors discussed the limitations of IoT devices especially in battery and computing resources and possible solutions are discussed. The authors in [29] presented a survey on security threats at different IoT layers. The authors discussed some security solutions such as blockchain, machine learning, edge and fog computing. Similarly, the authors in [30] presented a survey on IoT security issues. The authors presented a comprehensive analysis highlighting the security goals, attacks and solutions. The authors in [31] present an overview about different layered architectures of IoT and attacks regarding security from the perspective of layers. The authors also review mechanisms that provide solutions to these issues with their limitations. Furthermore, they suggested a new secure layered architecture of IoT to overcome these issues.

In general, these survey papers highlighted some state-of-the-art analysis of IoT and LPWAN security. However, the survey works do not cover most of the threats and security challenges that are attributed to LPWAN. Thus, this paper fills the research gap by bringing together a wide range of LPWAN security challenges, the methodologies employed in mitigating security attacks and research future directions. 

## 3. Overview of LPWAN

LPWAN is a wireless technology that have continued to gain enormous attention in recent times. The technology is specifically designed for long range communications (up to 50 km depending on the LPWAN device) [32,33,34,35]. LPWAN technologies are distinguished from other connectivity technologies as they have outstanding features which include low power consumption, low cost, bit rate, capacity and mobility [20]. Furthermore, they provide battery-efficient possibility targeting 10+ battery life, universal wide-area connectivity thereby allowing numerous M2M and IoT applications unlike popular short-range network devices such as Bluetooth, Wi-Fi, and ZigBee [36]. With regards to network topologies, most LPWAN technologies conventionally deploy single-hop networks based on star topology as it assists in preserving battery power, safe cost and it also compensate for communication range. Depending on the region and standards, LPWANs can operate in the unlicensed industrial, scientific, and medical (ISM) frequency bands 2.4 GHz, 868/915 MHz (Europe/North America), 433 MHz (Asia) etc. [32,37,38]. Some LPWAN can operate on the licensed frequencies [39]. Some of the general attributes and characteristics of LPWANs that make them suitable for IoT connectivity are discussed as follows. 

### 3.1. Low Power Consumption

Low power consumption is considered as one of the significant features attributed to LPWANs. LPWA networks devices are typically battery powered and are usually effective and reliable for a very long term without human interference. The authors in [13] explained that LPWANs have the capacity to reduce the amount of energy consumed by utilizing sleep mode. In such modes, the transceiver devices are responsive only when data are to be transmitted or received.

### 3.2. Wide or Extended Coverage

Wide coverage is also one of the paramount strongpoints for LPWAN mass deployments. The extended coverage range allows end devices to stay connected to base stations that are probably kilometers away. Thus, the network infrastructure deployment is suited for varieties of applications that require long range connectivity such as smart cities, power grid management, and smart agriculture [40] and applications such as underground pipelines where existing conventional communication technologies struggled in reaching. 

### 3.3. Scalability

Scalability is one of the core features of LPWAN. LPWAN technology are well known to support multiple devices connectivity in a scalable manner without compromising the quality of services. As it is envisaged that the number of connected devices in an IoT environment will reach billions and even surpass the cellular technology growth by 2025, it is expected that LPWAN technologies will play a key role in the future scheme of things. 

### 3.4. Security and Privacy

As numerous cyberattacks and intrusion menaces have continued to plague the deployment of wireless devices, security and privacy vulnerability concerns have been identified as a major challenge in IoT applications [15,41]. To avoid intrusion, security measures and features such as authentication, encryption (AES, RSA, etc.) and hardware security (e.g., tamper-proofing) are characteristics attributed to devices connected within LPWA network [42].

## 4. LPWAN Technologies

As of today, there are several LPWAN standards including SigFox, Long Range (LoRa), Weightless, DASH7, Narrow Band-IoT (NB-IoT), and Ingenu-RPMA in existence [43]. These existing LPWAN standards have individual feature attributes for addressing general IoT problems such as scalability, security, and power consumption. Also, the existing standards have several characteristics that may exist or may not exist in other similar standards [44]. This section briefly discusses some of the popular LPWAN technologies and their individual technical specifications especially in terms of physical layer and medium access control (MAC) layer specifications.

### 4.1. LoRa

Long Range (LoRa) is a LPWAN standard developed by LoRa Alliance [33,45]. LoRa operates in unlicensed sub-GHz ISM band. LoRa is a physical layer standard that operates based on chirp spread spectrum modulation whereby the modulation process is done by representing individual bit of payload information by multiple chirps of information [46]. For inference effect mitigation, LoRa standards use Forward Error Correction coding for controlling errors in transmission over reliable communication channels. 

**Figure 1:**
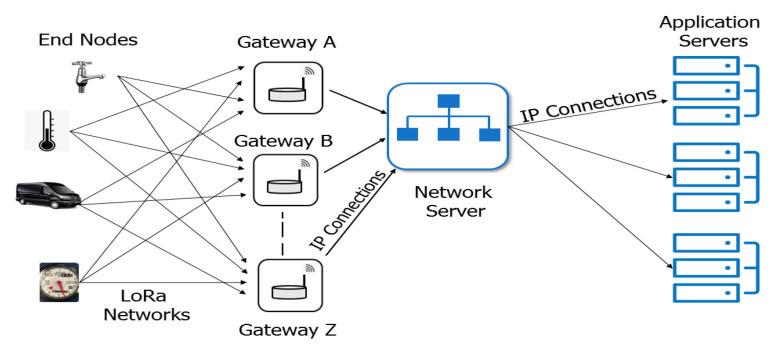
Structural architecture of a LoRaWAN [47].

The data rate allowed by the standard varies from 300 bps to 50 kbps, values subjected to the spreading factor (SF) and channel bandwidth settings. LoRa deploys a quasi-orthogonal transmission method with different SFs and they allow multiple transmissions with different SFs at the same time. The standard use ALOHA principle for channel access and like most LPWAN, they mostly use the single-hop technology. Figure 1 presents the structural architecture of a typical LoRaWAN. 

As shown in Figure 1, a typical architectural framework of a LoRaWAN comprises of end-devices, gateways, network server and application servers. The end devices are connected to the network server through a LoRa gateway usually using a star-to-star topology. Generally, the end devices are connected to multiple gateways using single hop packet transfer [10]. Afterwards, the gateways forward the received packets to the network server through a back-haul interface which may be ethernet, Wi-Fi, cellular etc. When the network server receives the packets, it performs important role such as network and security management, verification of end-devices addresses, and removal of packet redundancies [21]. Finally, the network server forwards the messages to the application servers for decryption and the initiation of new actions. 

### 4.2. Sigfox

**Figure 2:**
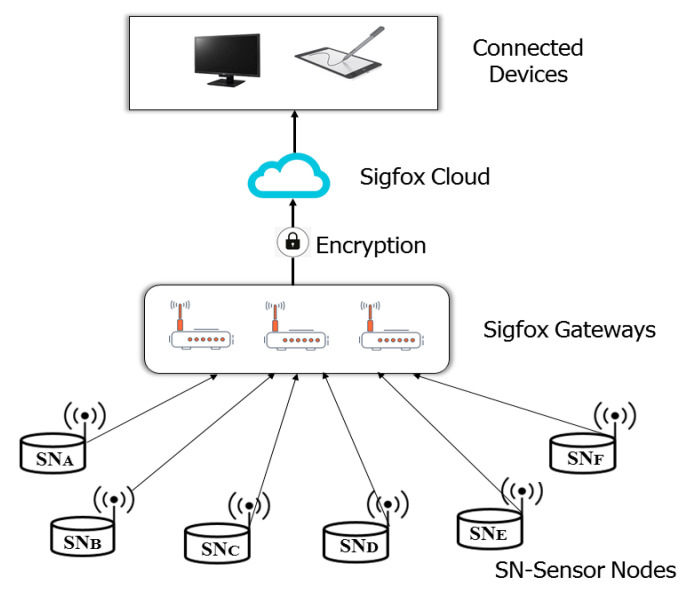
Architecture of a typical SigFox network [49].

Sigfox [48] is a LPWAN technology that employs ultra-narrowband (UNB) modulation techniques. The modulation technique allows the receiver to only listen in a tiny slice of the spectrum, in order to minimize noise interference. They have the capacity to achieve a range between 10–50 km with minimal noise interference. The standard deploys differential binary phase-shift keying (DBPSK) and the Gaussian frequency shift keying which allows connectivity on the ISM frequency band. Sigfox operates on 902 MHz and 868 MHz in USA and Europe respectively [49]. Initial Sigfox release was only one directional communication system; however, the recent release supports a bidirectional communication. Sigfox communication standards support up to 140 uplink messages a day, each of which can carry a payload of 12 octets at a data rate of up to 100 bits per second. Figure 2 depicts the architecture of a typical Sigfox standard. Similar to LoRaWAN, Sigfox also deploy ALOHA principle for channel access and the architectural framework of a typical Sigfox network also consist of various end nodes, Sigfox gateways, Sigfox cloud and Application server. The network is based on one-hop star topology. The end nodes can be connected to gateway using star topology to relay messages to the gateways. The individual gateway forwards the received data to the Sigfox cloud using secure IP connections. The cloud is responsible for data management and data processing before sending it to the application server for further processing [50].

### 4.3. Weightless

Weightless is one of the newest LPWAN technology. The standards are launched by Weightless Special Interest Group [51]. The group released three open LPWAN standard namely: Weightless-N, Weightless-P and Weightless-W. For modulation, the three standards utilize Gaussian Minimum Shift Keying and Quadrature Phase Shift Keying modulation techniques; and 16-Quadrature Amplitude Modulation techniques respectively [47]. Furthermore, another major difference between the three standards is that Weightless W adopts TV whitespace, Weightless N support one-way communication and Weightless P support bidirectional narrowband technology. Also, Weightless P have the capacity to operate in both the licensed and unlicensed ISM frequency bands. 

### 4.4. Ingenu RPMA

The RPMA technology is proposed by INGENU. Different from other popular similar standards such as LoRa and SigFox, RPMA do not operate in the sub-band frequencies, it operates in the global 2.4 GHz ISM band instead. Also, the standard allows wider coverage and higher energy consumption compared to similar LPWAN standards. With regards to the data rate allowed, Igenu RPMA allows up to 624 kbps on the uplink (UL) and 156 kpbs on the downlink (DL). For modulation, Ingenu RPMA uses the Direct Sequence Spread Spectrum (DSSS) techniques. For MAC, Ingenu RPMA uses Code Division Multiple access (CDMA). One of the strongest points of RPMA is its scalability which is alleged to be unlimited well known.

Well known for their robustness and long-range communication, the Ingenu 2.0 have been identified as the best IoT technology (RPMA) in the market today for the non-licensed spectrum and the standard is widely used in oil and gas field automations. 

### 4.5. Narrowband Internet of Things

NB-IoT is a LPWAN radio technology standard introduced by 3GPP to enable a wide range of cellular devices and services. As a 3GPP standard, several mobile equipment, chipsets and module manufacturers supports NB-IoT and the LPWAN standard can co-exist with 2G, 3G, and 4G networks. Furthermore, the standard benefits from distinctive mobile network features which include strong support for user identity confidentiality, authentication, and integrity. The standard enables strong connection density and they have admirable battery life. NB-IoT operate at a frequency bandwidth of 700 MHz, 800 MHz, and 900 MHz. NB-IoT is based on the characteristics of long-term evolution (LTE) protocol which enables its integration and easy deployment with LTE network. NB-IoT use Orthogonal Frequency-Division Multiple Access for downlink and Single-Carrier Frequency-Division Multiple Access [52]. With regards to the data rate allowed, NB-IoT allows up to 158.5 kbps on the UL and 106 kbps on the DL. The transmission power of NB-IoT is +23 dB and the maximum payload size for each message is 1600 bytes. [53]. Table 1 presents an analytical comparison of LPWAN technologies and their technical characteristics.

## 5. LPWAN Security Analysis

The rise of IoT and LPWAN deployment have continued to be threatened by various security and privacy issues [17,47]. By LPWAN security, we mean the level of resistance to, or protection of the entire applications, data, and infrastructures in the LPWAN network. Majority of the LPWAN architectural infrastructures including end devices, gateways and network servers have been identified as easy targets for sabotages and intrusions due to various reasons such as the heterogeneity of devices, open nature of connecting devices to the Internet, scalability and their accessibility remotely. Also, once an integral layer such as the network layer is sabotaged, intruders can easily take control and mischievously use any of the end devices or even the whole network devices. Just like other IoT technologies, the pervasive nature of the information being produced, processed, transmitted and stored within LPWANs have significant implications on the networks’ security. Prominent information security requirements confidentiality, integrity and availability otherwise known as “CIA triad” are key necessities to guarantee security for LPWA networks [55].

### 5.1. Confidentiality

Confidentiality requirements refer to a situation whereby only the senders and the recipients of packets can access transmitted data among nodes i.e., sensitive data should be protected from unauthorized accesses [56,57]. Two of the most common confidentiality attacks is the man-in-the-middle (MITM) attacks and compromised key attack. MITM refers to an attack whereby intruders furtively alter the communications between two nodes who assume there is no third-party involved in their conservation. A typical example of the MITM attack is the popular eavesdropping attack. Eavesdropping attack refers to a situation whereby intruder passively listen to the network communication over compromised communication links to capture or gain access to private information ranging from access codes to passwords. On the other hand, compromised key attack refers to a situation whereby attackers use stolen keys to gain admission into a network. The stolen accessed key is referred to as “compromised key”. Compromised key allows intruders to decrypt, modify or alter data that are being sent, and to access communications [58]. 

With regards to LPWAN confidentiality menaces, various studies in the literature have discussed the confidentiality challenges, effects and most significantly, proposed varieties of solutions. Describing the issue of key management in LoRaWAN, the authors in [59] explained that ideally, the network server is responsible for creating session keys (i.e., network key and the application key) in LoRaWAN v 1.0, and the authors identified unauthorized access into the network server as a serious threat. The authors proposed a public key infrastructure (PKI) scheme as a trusted third-party solution to the key crisis problem. Despite the success achieved from the proposed solution, the authors acknowledged that the procedure can be computationally inept, complex, and time-consuming as the communication overhead is increased due to the newly-added third party. Similarly, the authors in [60] proposed a LoRaWAN architecture enhancement solution using several AES-128 encryption keys at the network layer and application layer for data authentication and privacy. The authors deployed a reputation system that utilizes proxy nodes to reduce the complex computing in the constrained node side thereby providing interoperability and adequate security of message exchanges between end nodes.

In another compromised key attack security study involving LoRaWAN, the authors in [61] proposed an Ephemeral Diffie–Hellman Over COSE (EDHOC) based lightweight and authentication approach that uses a cryptographic material for updating LoRaWAN session keys at the application layer. The authors in [21] also proposed a dual key-based activation scheme for mitigating LoRaWAN key update security problems. The approach involves utilizing network key and application key in performing initial join procedure and the second join procedure for a real-world testbed. For additional security, the author proposed the generation of each session key independently at each layer and there are no additional third-party entities involved. For the strengthening of session key derivation, the authors in [62] proposed a Rabbit stream cipher-based Key Derivation Function (KDF) scheme for updating the root key. The two-step KDF offers high computing efficacy and it provides suitable randomness of generated keys.

### 5.2. Integrity

Data integrity refers to the preservation of network data accuracy, completeness and reliability [63]. As explained by authors in [64], data integrity aims to prevent unintentional or intentional manipulation, alteration, deletion, or modification of network information and commands by unauthorized intruders. Typical integrity attacks include replay attack, sybil attack, and wormhole attack. A replay attack can be described as an attack whereby a valid packet transmission is maliciously manipulated [65]. The authors in [65] explained that replay attacks are perpetrated by intruders who capture network traffic and communicate with authorized users/nodes while acting as a legitimate node. With regards to wormhole attack, [66] described the attack as a situation whereby an intruder receives network packets at one location, forwards the retrieved packets to another node, and continues to replay the packets within the entire network. Furthermore, wormhole attack are closely tied to other attacks such as eavesdropping and replay attacks. In [15] the authors explained that a wormhole attack can be performed using two devices: Sniffer and jammer. The sniffers capture the network packets and decide on whether to jam the packets. Once a decision to jam has been made, a notification is sent to the jammer to jam the packet immediately. To prevent wormhole attack, the authors in [67] proposed that the two devices (sniffer and jammer) must be kept separate to prevent sniffer from recording the original message and to circumvent jamming of the messages. In sybil attack, a malicious device from intruders illegally creates numerous identities in the network and the identities are used to gain a disproportionately large influence within the network [68]. 

The analysis of LPWAN data integrity have been a major topic in the literature as major LPWAN standards are known to be highly vulnerable to integrity attacks. In an analysis of LPWAN integrity vulnerabilities, the authors in [15,69] cautioned that Sigfox is highly vulnerable to replay attacks and they strongly advised against its deployment for critical applications. However, the authors in [69] advised that better replay protection must be integrated at a higher layer by end users, thus minimizing the already small payload size. Similarly, the authors in [17] explained that Weightless-P network are also prone to replay and wormhole attacks. 

Various works in the literature have discussed numerous security solutions for LPWAN integrity attacks’ mitigations. While presenting a comprehensive analysis on LoRaWAN security issues, the authors in [70] proposed a dual option (default option and security enhanced option) security protocol for preventing replay attack. The security protocol was developed to support mutual authentication, secret key exchange, perfect forward secrecy, and end-to-end security between node devices. Similarly, to establish a secured end to end LPWAN communication, the authors in [20] proposed a replay attack mitigation scheme involving two different AES keys. The authors used a frame counter which involves two different 128-bit AES session keys for upstream and downstream messages exchange, respectively, for the blockage of repeated transmission of packets. For robustness, the authors further encrypted each message using the XOR operation with the corresponding key. Also, the authors in [71] presented a replay attack scenario that typically occur at the join request transfer process. In a detailed mitigation solution to the menace, the authors proposed the deployment of sniffed join request messages approach. In another security assessment study, the authors in [22] proposed an AES-128 based Secure Low Power Communication (SeLPC) method for enhancing the security level of LoRaWAN communication by periodically updating encryption key (AppSKey) and lookup table (D-Box) on both end-devices and application-server sides. In a similar LoRaWAN v1.0 replay attack study, the authors in [72] initially analyze the typical approach that attackers used to implement the replay attack specifically as “replay of join accept message” and “harvest of join message”. For its mitigation, the authors proposed an increment in the size of Devnonce and AppNounce value with no repetition. DevNonce is a random number generated by the end node. It is employed for preventing replay attacks and for generating session keys. Likewise, the author in [73] also propose the DevNonce approach of preventing replay attack. The author suggested that network servers should store all DevNonces used in the previous join procedure in order to prevent replay attack. Furthermore, the author hypothetically analyzed DevNonce method especially in a case whereby end nodes are unavailable. In such case, the authors proposed the increment of the size of the DevNonce field to 24 or 32 bits. In another study, the authors in [74] presented a solution for bit flipping attacks in LoRaWAN. The authors proposed a shuffling method that is performed by end devices and it aims at preventing attackers from identifying positions of message field from bit-flipping attacks. 

### 5.3. Availability

Availability refers to a situation whereby network resources are always available for authorized user when required [57,58]. Data availability prevents bottleneck situations which hinders information flow. The most prominent availability menace is Denial of Service (DoS) attacks. DoS attack is described as an attempt by malicious attackers to consume network resources or bandwidth [55]. The most common DoS attack include DNS flood, Internet Control Message Protocol (ICMP) broadcast and SYN flood [75,76]. The authors in [67] highlighted jamming attack (a subset of DoS attack) as a major security issue in LoRa and LoRaWAN. With regards to LPWAN availability menaces mitigations, several encryption models, IDSs and other security measures have been proposed in the literature. IDS are security systems or models that monitor and collect information from various infrastructures within a network and analyzes the data in an attempt to detect malicious events that threaten the network. The data collected overtime aids the network and network administrators in preparing for, and deal with intrusion and intrusion attempts on their networks. The major types of IDS are network-based and host-based. While host-based IDS deal with internal monitoring whereby it collects information about activities on a particular single host that is well known to be susceptible to possible attacks, network-based IDS collect information from the entire network traffic stream. For more details on IDSs, the authors refer readers to [77,78,79,80,81]. With regards to IoT security, various approaches including machine learning and deep learning models have been proposed for IDSs in the literature. The authors in [82] proposed an IDS using an ensemble of boosted and bagged trees, subspace discriminant and RUS boosted trees for detecting routing attacks against IPv6 Routing Protocol. Testing their algorithms on RPL-NIDDS17 dataset, the authors acknowledged the ensemble of boosted trees with an accuracy of 94.5% outperformed the other models. Similarly, the authors in [83] combined network virtualization with deep learning algorithms as IDS for detecting various prominent attacks including DoS on IoT network. The authors in [84] also proposed an IDS for the detection of DDoS attacks in IoT networks using the hybridization of multi-objective optimization algorithms and deep learning algorithms. Also, [85] proposed an IDS using multi-layered perceptron neural network for detecting DoS attacks in IoT networks. The proposed scheme achieved an accuracy of 92.84% in successfully detecting various form of DDoS/DoS attacks.

Despite the numerous varieties of IDS works on the general IoT domain, only a few studies in the literature have specifically considered IDSs for mitigating the various security challenges in LPWANs. The authors in [19] proposed a Kullback Leibler Divergence (KLD) and Hamming Distance (HD) based IDS for detecting jamming attacks in a developed LoRaWAN testbed. In the developed experimental testbed that is made up of hardware devices which includes raspberry pi 2, Arduino uno and LoRa SX1272 mbed shield that are used as gateway and end node devices. In the study, a network server that is written in python was programmed by the authors. In a similar mitigation study, the authors in [86] addressed the issue of lack of forward secrecy, flaw in delegation of join procedure in case of fall-back, and limitations in replay protection that are peculiar with LoRaWAN v1.1. The authors proposed countermeasures that tackles the application data integrity and confidentiality violations in case of join procedure delegation and malicious network server.

Apart from the cyber security issue which covers the application layer and network layer of LPWA networks, security issues and attacks can be perpetrated at LPWAN physical layer as well. As IoT and LPWAN devices are extensively deployed in an open environment, these have allowed larger attack surfaces and physical access to network hardware devices such as sensors, nodes and actuators. Physical attacks which can be committed by disgruntled employees/ex-employees, agents, protesters, thieves, etc., aim to tamper, manipulate, expose, delete, or acquire access into LPWA networks, thereby inducing integrity attacks include node tampering and side-channel attacks on the network. In node tampering, the intruder can capture an end device and initiate the collection of vital information from it thereby compromising the whole network. Furthermore, the intruder can destroy or replace the node device and security keys can be stolen and compromised [87,88]. On the other hand, in a side-channel attack, an attacker can snoop or exploit vulnerabilities from the implementation of the network hardware. In such attacks, timing information, electromagnetic leaks or even sound wave, etc. can be snooped on, in order to monitor and gather information on network events as well as network keys. The information collected can then be used to compromise the network. As IoT devices including LPWAN end nodes are mostly Complementary Metal-Oxide-Semiconductor (CMOS) based, they are highly vulnerable to this form of attack. To mitigate these attacks, the use of various hardware mechanisms which can ensure proper authentication and access control mechanisms, tamper resistance and other security mechanisms such as the secured elements (SE) proposed in [89,90,91,92,93,94] are particularly important for IoT devices. Hardware-based secure elements can provide a high level of security required by various IoT applications. SE is a microprocessor chip which can store confidential information. The SE architecture is equipped with hardware devices component, such as a cryptographic co-processor, secure random number generator, secure memory, and tamper-proofing technology. Apart from physical attacks, natural disasters, wear and tears can also disrupt the normal operation or initiate the loss of LPWAN components such as end nodes such as sensors or actuators.

## 6. Comparison and Discussion

In the literature, numerous research works have been done on IoT and LPWAN at large. However, there have been relatively limited research works focusing on LPWAN security assessment and attack countermeasures. Most of the LPWAN security research works in the literature focused mainly on analyzing the threats and vulnerabilities of LPWAN technologies with less details on proffering possible solutions to the menaces. In Table 2, we summarized some recent research works that discuss the different LPWAN security threats and the solutions proposed. Table 2 presents the comparison of different LPWAN security challenges discussed by various authors in the literature, the methodology deployed by the authors, their strengths and drawbacks are briefly summarized. Most of the solutions highlighted by the various authors in the table have not been experimentally tested to prove their feasibility in real world attack mitigation. Thus, there is still a wide gap in building effective security framework for LPWANs.

## 7. Research Challenges and Recommendations for Future Works

Despite the promising benefits and the brilliant forecasted future of LPWANs, there are still significant security challenges in current security works, in the development and deployment of existing standard, etc., that call for further investigations. Most of the currently existing security measures and studies majorly focus on cryptographic algorithms and key management problems. Despite the successes achieved, series of security challenges are yet arising on daily basis as networks are still susceptible to technical challenges such as intrusions. Thus, it is highly important to provide efficient security measures that can quickly identify, detect, isolate compromised devices. In this section, we discuss some of the significant challenges that are posing major threats to the current deployment of LPWAN and some recommendations for the future. 

### 7.1. Key Management and Storage

Key management and storage have always been a major issue in LPWAN. In LPWAN, secret keys are typically stored in electrically erasable programmable ROM (EEPROM) of the nodes. The EEPROMs are highly vulnerable to various attacks such as side-channel attacks. Addressing the issue of key storage, the authors in [95] explained that if the application server that holds all the secret key is compromised, the communication between the entire nodes in the network can be compromised. In order to ensure that secured communication within the networks is achieved, adequate security measures such as proper and fast encryption, authentication measures, tamper proofing, and secure elements (SEs) are some of the methods to be considered. 

### 7.2. Encryption Factors

One of the key factors thwarting the full realization of the LPWAN is the problem of inefficient encryption measures. The encryption in most existing LPWAN standards provides a weak level of data confidentiality and integrity. The use of symmetric encryption is not secured as it uses single key to execute encryption process. Although some LPWAN standards use asymmetric encryption like RSA and strong encryption methods such as AES, better and faster encryption methods for LPWAN should be core interest of future research works [20]. 

### 7.3. Bootstrapping and Authentication Issue

The effective and efficient control of LPWAN entry of nodes is also one of the major LPWAN security topics. The effective deployment of LPWAN requires proper identification and verification of legitimate devices especially the end node devices. Generally, IoT use authentication servers mostly via network access protocols such as Protocol for Carrying Authentication for Network Access (PANA) [96] for a node to join a network. In a typical LPWAN setup with inadequate authentication measures in place, an attacker can modify encrypted payload without the application server being able to notice the change. 

### 7.4. Jamming

Just like any other cyber domain, jamming is a major challenge for LPWAN. The proximity of intruders to end node devices have constituted to the huge probability of jamming attacks in LPWAN [69]. Thus, effective and innovative techniques such as IDSs should be a core interest of future research efforts in order to mitigate jamming in LPWAN.

### 7.5. Compromised IoT Device and Open Environment

Since LPWANs support enormous number of IoT devices, the deployment of these devices that operate in an open environment makes them vulnerable to varieties of security threats. Therefore, effective security schemes to detect, isolate and classify malicious nodes such as intrusion detection systems [19] and key management mechanisms for IoT devices should be considered for increased communication reliability and improved quality of service. Also, ensuring the physical layer security issues are addressed such as deploying efficient tamper proofing protections.

### 7.6. Untrusted Gateways

Gateways are transparent bridges between end nodes and network server. They create communication between end nodes and the server. In most of the implementation scenarios, a few number are deployed. The deployment of the gateways in an open environment makes them untrusted devices. Since the gateway is communicating directly with the network, if an intruder gains access to a gateway, the data passing through it can be easily recorded and even manipulated. The manipulation of these gateways can lead to higher power consumption of end devices which can breakdown the end devices. Also, the communication between the end devices and the rest of the network can be destroyed. To provide secure data transmission, an authentication mechanism for the gateways is necessary in order to prevent the network from attacks [97]. 

## 8. Conclusions

LPWAN is one of the most adopted technologies and arguably the fastest growing connectivity standards in IoT application. However, the LPWAN technologies has a major challenge in the form of security and privacy vulnerabilities. Through various means and loopholes at the various layers and infrastructures within the networks, intruders can attack and create undesirable events that can compromise the entire network. To address these problems, this paper presents a comprehensive review of the most recent approaches deployed in addressing the major LPWAN security and privacy menaces. The paper presents several security methodologies proposed in recent literature and the paper compares their respective advantages and limitations. Furthermore, the paper discussed security requirements that need to be evaluated and considered in designing and implementing secured LPWA Networks. Finally, the paper highlight some of the general LPWAN research challenges that require attention in future works. 

## Figures and Tables

**Table 1:** Some prominent low power wide area network (LPWAN) technologies and their technical features [15,32,37,47,54].

LPWAN Type	Modulation	Freq. Band	Security/Encryption	Occupied Bandwidth	MAC	Range	Max Data Rate
LoRa	CSS/FSK	Sub-GHz ISM: Europe (868 MHz, 433 MHz) USA (915 MHz)	AES 128 bit	250 kHz, 500 kHz and 125 kHz	ALOHA	Urban-Rural 5–20 km	50 kbps
Sigfox	UNB (DBPSK and GFSK)	Sub-GHz ISM: Europe (868 MHz) USA (902 MHz)	AES + no key OTA emission	UL (100–600 Hz) DL (1.5 kHz)	ALOHA R-FDMA	Urban-Rural 10–50 km	UL (100 bps) DL (600 bps)
NB-IoT	QPSK	LTE frequency Bands	2048-Bit RSA	200 kHz	OFDMA	15 km	UL (158.5 kbps) DL (106 kbps)
DASH7	GFSK	915, 433 and 868 MHz	AES 128	25 and 200 kHz		0–5 km.	167 kbps
Ingenu-RPMA	DSSS	2.4 GHz ISM	AES 128, 16B Hash	1 MHz	CDMA	Urban15 km, 500 km LoS	UL (624 kbps) DL (156 kbps)
Weightless	16QAM, offset-BPSK, QPSK, GMSK and DBPSK	Numerous Bands (sub-GHz)	AES 128/256 Bit	200 Hz - 12.5 kHz	FHMA with TDD	Up to 5 km	100 kbps

**Table 2:** A Comparison of Different Scheme Employed to Prevent LPWAN Attack.

Refs.	Security Threat(s) Addressed	Security Requirements	Brief Summary of Approach, Highlight Strength and Limitation
[62]	Problem of key updates	Confidentiality	Proposed the use of a root key update scheme for reinforcing the session key derivation security. **Strength:** Requires fewer computing resources. Offers suitable randomness of the generated updated key.
[20]	Replay attack	Integrity	For the blockage of repeated transmission of packets, a frame counter which involves two different 128-bit AES keys: AppSKey and NwkSKey for upstream and downstream messages exchange was proposed.
[59]	Key management security flaws	Confidentiality	A trusted third-party PKI (scheme) was proposed. **Strength:** Strong key management and distribution. **Limitation:** High computation involved due to the involvement of a third party. Complex join produce.
[60]	Key management issue	Confidentiality	Several AES-128 encryption keys at the network layer and application layer was used for data authentication and privacy respectively.
[61]	Compromised key	Confidentiality	Ephemeral Diffie–Hellman Over COSE (EDHOC) approach that uses a cryptographic material derived at the application layer for updating LoRaWAN session keys is proposed. **Strength:** Low computational cost and flexibility in session keys updates.
[21]	Problem of key updates	Confidentiality	Proposed a dual key-based activation scheme for LoRaWAN security solution. NwkSKey and AppSKey was used in performing initial join procedure and the session key created in the initial join procedure is used for second join procedure. **Strength:** No third party involved. Secured connectivity between end devices and application server. **Limitation:** Perfect forward secrecy is not guaranteed.
[74]	Bit flipping attack	Integrity	Proposed a shuffling method to prevent bit flipping attack. **Strength:** Prevent attackers from identifying positions of message field from bit-flipping attacks. **Limitation:** Not suitable for devices with low power and low resources.
[70]	Replay attack	Integrity	Proposed a security protocol that comprises of a dual option (default option and security enhanced option) for preventing intruders from breaking the end-to-end security between a device and the application server. **Strength:** Supports mutual authentication, secret key exchange, perfect forward secrecy and end-to-end security.
[22]	Replay attack	Integrity	Proposed an AES-128 based Secure Low Power Communication (SeLPC) method to boost the security level of LoRaWAN communication. **Strength:** Efficient power consumption.
[71]	Replay attack	Integrity	Used sniffed join request messages to prevent replay attack. **Strength:** Fully support secure key exchange. **Limitation:** The approach does not support the perfect forward secrecy nor end-to-end security.
[72]	Replay and Decrypt attack	Integrity	Proposed the increment in the size of DevNonce and AppNonce value with no repetition.
[73]	Replay attack	Integrity	Network server store all DevNonces used in the previous join procedure in order to prevent the attack.
[19]	Jamming attack	Availability	IDS that is based on KLD and HD was used for detecting jamming attacks in a LoRaWAN Network. **Strength:** Ability to detect and respond quickly to anomalous behavior. Ability to detect new forms of attacks which might deviate from the normal behavior. **Limitation:** Prone to false positives.
[17]	Replay and Wormhole attacks.	Integrity	Used data counter to prevent the attacks.
[86]	DoS attack	Availability	The Appskey derivation mechanism need to be changed and a special case for join procedure delegation must be introduced.

## Abbreviations

Tables presents all the acronyms used in the paper. Definitions of all acronyms mentioned in the paper.

AES	Advanced Encryption Standard
AppSkey	Application Key
BPSK	Binary Phase-Shift Keying
CDMA	Code Division Multiple access
CKA	Compromised Key Attack
CSS	Chirp-spread-spectrum
DBPSK	Differential Binary Phase Shift Keying
DL	Downlink
DoS	Denial of Service
DSSS	Direct Sequence Spread Spectrum
EDHOC	Ephemeral Diffie-Hellman Over COSE
EEPROM	Erasable Programmable ROM
FEC	Forward Error Correction
FHMA	Frequency Hop Multiple Access
FSK	Frequency-shift keying
GFSK	Gaussian Frequency-Shift Keying
GMSK	Gaussian Minimum Shift Keying
HD	Hamming Distance
IDS	Intrusion Detection System
IoT	Internet of Things
ICMP	Internet Control Protocol
ICT	Information Communication and Technology
ISM	Industrial Scientific and Medical
Kbps	Kilobits Per Second
KDF	Key Derivation Function
KLD	Kullback Leibler Divergence
LoRa	Long-Range
LoRaWAN	Long Range Wide Area Network
LoS	Line of Sight
LPWAN	Low Power Wide Area Network
MITM	Man-in-the-middle
M2M	Machine-to-Machine
NB-IoT	Narrow Band-Internet of Thing
NwkSkey	Network Key
OFDMA	Orthogonal Frequency Division Multiple Access
OTA	Over-The-Air
PKI	Public Key Infrastructure
QPSK	Quadrature Phase Shift Keying
RPMA	Random Phase Multiple Access
RSA	Rivest–Shamir–Adleman
R-FDMA	Random Frequency Division Multiple Access
SE	Secure Elements
SeLPC	Secure Low Power Communication
TDD	Time Division Duplex
UL	Uplink
UNB	Ultra-Narrowband
